# Effect of Induction Hardening Conditions on Effective Case Depth, Microstructure, and Tribological Properties of C50 Steel Inner Races in Constant-Velocity Joints

**DOI:** 10.3390/ma19194257

**Published:** 2026-10-08

**Authors:** Krzysztof Włoch, Sylwia Wencel, Piotr Szablewski

**Affiliations:** 1 Neapco Europe Sp. z o.o., ul. Kaliska 72, 46-320 Praszka, Poland; 2 Faculty of Production Engineering and Materials Technology, Czestochowa University of Technology, Av. Armii Krajowej 19, 42-201 Czestochowa, Poland; sylwia.wencel@pcz.pl; 3 Institute of Mechanical Engineering, University of Kalisz, Wojciecha Boguslawskiego 2, 62-800 Kalisz, Poland; p.szablewski@uniwersytetkaliski.edu.pl

**Keywords:** induction hardening, scanning, single shot, effective case depth, martensitic microstructure, hardness profile, tribological behavior, CV joint inner race

## Abstract

Increasing torque density and dynamic loading conditions in modern automotive drivetrains, particularly in electric vehicles, impose stringent requirements on the surface integrity of constant-velocity (CV) joint components. Induction hardening of medium-carbon steels is a potential alternative to conventional carburizing; however, the influence of hardening strategy on hardened layer characteristics and functional performance remains insufficiently explored for CV joint inner races with complex geometry. In this study, industrial-scale induction hardening trials were performed on C50 steel inner races using two processing strategies: single-shot and scanning. An industrial-scale comparative study was conducted to evaluate hardness distribution, effective case depth (EDH_500_HV), microstructure, and tribological behavior under representative single-shot and scanning processing conditions. Hardness reached the target range of 653–772 HV1 (≈58–63 HRC) with both strategies. However, clear differences in effective case depth and microstructural homogeneity were observed. The scanning strategy showed a more uniform near-surface hardness distribution and lower variability in effective case depth compared with single-shot hardening. Tribological testing under dry reciprocating sliding conditions revealed comparable friction coefficients, while variations in wear scar morphology were associated with differences in microstructural continuity and transformation uniformity. The results establish a relationship between induction energy input, phase transformation behavior, case depth control, and functional wear response, providing an industrially applicable framework for process optimization of C50 steel CV joint inner races.

## 1. Introduction

Constant-velocity (CV) joints are critical drivetrain components that enable torque transmission under variable articulation angles while maintaining constant angular velocity between input and output shafts. In modern automotive applications, particularly in electrified powertrains, drivetrain components are exposed to fundamentally different loading characteristics compared with conventional internal combustion engine (ICE) systems.

Electric motors deliver high torque from standstill and maintain a broad constant-torque region over a wide range of speeds [1]. In contrast, ICEs typically reach peak torque at intermediate speeds, with significantly lower torque available at low rotational speeds. As a result, electrified drivetrains impose sustained high-contact loads at low and medium rotational speeds, increasing the mechanical demand on CV joint raceways.

**Figure 1:**
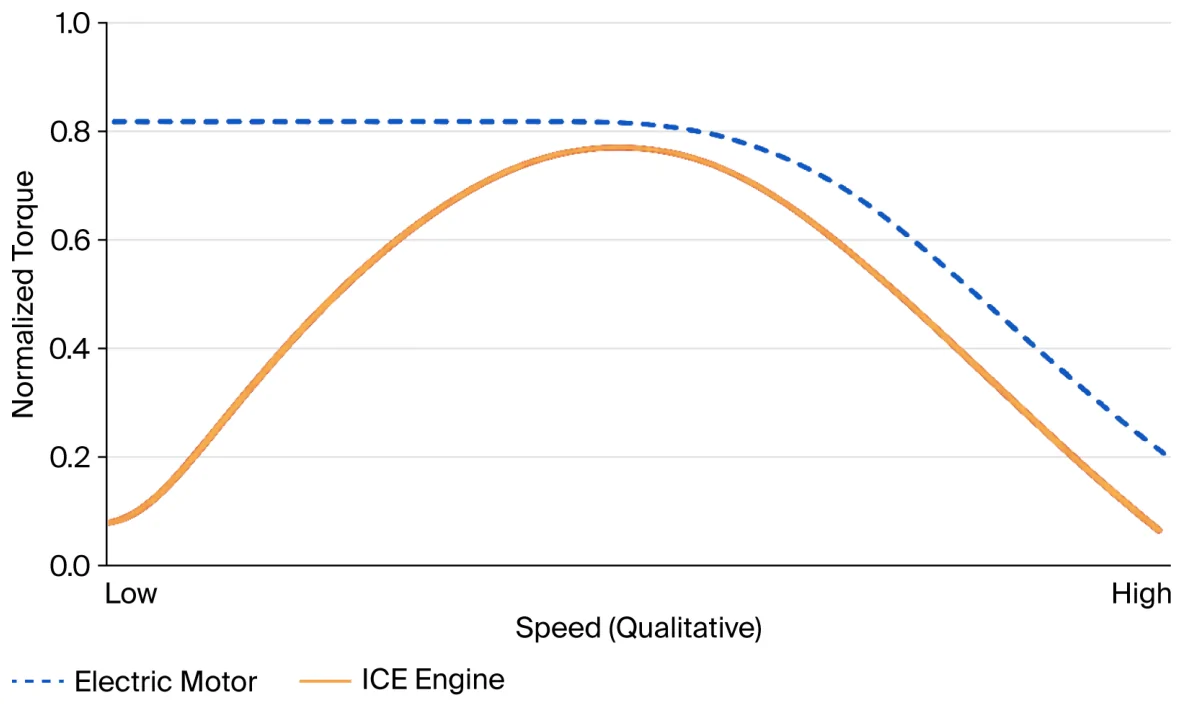
Conceptual schematic representation of normalized torque-speed characteristics for electric motors and internal combustion engines.

Figure 1 shows that electric powertrains operate with elevated torque at low speeds, directly influencing Hertzian contact stresses in rolling-sliding interfaces. Since contact pressure scales with transmitted torque, elevated low-speed torque increases the maximum Hertzian pressure and subsurface shear stress amplitude. In ball-type CV joints, the functional raceways of inner and outer rings are subjected to combined rolling-sliding contact under cyclic Hertzian pressures that may locally exceed 2 GPa, accompanied by tangential traction forces and localized micro-slip [2,3]. The resulting stress state is characterized by a maximum subsurface shear stress typically located at 0.5–1.0 mm below the contact surface, depending on contact geometry and load level [2,3].

Consequently, the hardened layer depth and microstructural homogeneity must be precisely engineered to ensure that the depth of maximum shear stress remains within the martensitic zone, thereby preventing subsurface fatigue crack initiation and spalling [3].

Traditionally, CV joint components are manufactured from low-carbon alloy steels subjected to carburizing followed by quenching and tempering [4,5]. Carburized steels provide a hard, wear-resistant surface layer (58–62 HRC) and a tough core, enabling favorable compressive residual stresses and high rolling contact fatigue resistance [6]. However, carburizing is associated with long cycle times, high energy consumption, global thermal exposure, dimensional distortion, and significant post-heat-treatment machining requirements [7,8]. These drawbacks are particularly critical for high-volume production and geometrically complex components such as CV joint inner races, where dimensional precision directly affects noise-vibration-harshness (NVH) performance.

Induction hardening of medium-carbon steels represents a potential alternative due to its localized heat input, reduced thermal distortion, and significantly shorter processing times [9,10]. Induction treatment enables selective hardening of functional surfaces while maintaining a ferrite-pearlite core, potentially improving dimensional stability and reducing manufacturing costs. Medium-carbon grades such as C45, C50, and 42CrMo4 have been investigated for use in automotive applications, including shafts, gears, and bearing components [11,12,13]. Among these, C50 steel offers a favorable balance between hardenability and microstructural uniformity in the as-delivered condition, promoting homogeneous austenitization during rapid induction heating.

The microstructural evolution during induction hardening is governed by heating rate, peak temperature, austenitization time, and cooling intensity [14,15]. Rapid heating may suppress diffusion processes, leading to non-equilibrium austenite formation and heterogeneous martensitic transformation, particularly in geometrically complex regions [16]. In addition, induction parameters influence residual stress distribution, which plays a critical role in rolling contact fatigue performance [17,18].

Effective case depth (EDH), commonly defined using a hardness threshold such as 500 HV1, is therefore a key design parameter in contact-loaded components [19]. Insufficient case depth may shift the maximum subsurface shear stress into a softer region, reducing fatigue life, whereas excessive case depth increases energy input and risk of distortion [6,19]. The precise control of thermal penetration and transformation kinetics is thus essential.

Two principal induction hardening strategies are commonly applied to rotational components: single-shot heating and scanning (progressive) heating [20]. In the single-shot strategy, the entire functional zone is heated simultaneously, resulting in rapid energy input and potentially deeper thermal penetration. In contrast, scanning involves the controlled movement of the coil along the component surface, allowing control of linear energy input and localized thermal gradients.

Although both single-shot and scanning induction hardening are well established, published investigations have predominantly focused on shafts, cylindrical specimens, and other comparatively simple geometries [8,11,12,13,21]. Taken together, these studies show that induction-hardening conditions and associated process parameters influence thermal penetration, hardness distribution, microstructural development, and the resulting hardened-layer characteristics. More recent investigations of progressive induction hardening have further shown that scanning parameters can significantly affect case depth, residual-stress distributions, microstructure, and wear behavior [22,23]. However, direct experimental comparisons of single-shot and scanning strategies for geometrically complex CV joint inner races remain limited. Consequently, the combined influence of hardening strategy on effective case-depth control, hardness distribution, microstructural characteristics, and functional tribological behavior relevant to CV joint contact conditions is not yet well established for this component geometry.

The increasing torque demands in electrified drivetrains and the need for energy-efficient manufacturing routes mean that a detailed understanding is required of the relationship between induction energy input, microstructure evolution, effective case depth, and tribological response in CV joint geometry.

Therefore, the aim of this study was to comparatively evaluate single-shot and scanning induction hardening strategies for C50 steel CV joint inner races. Specifically, the study investigated their effects on effective case depth, hardness distribution, microstructural characteristics, and tribological behavior, with the objective of identifying the strategy providing the most suitable combination of hardened-layer characteristics and functional performance.

## 2. Materials and Methods

### 2.1. Material

We investigated a medium-carbon non-alloy steel at grade C50 (EN 10083-2) [24], which has previously been identified as a suitable candidate for induction-hardened CV joint inner race applications based on comparative microstructural and stereological analysis. In a previous comparative stereological study [25], C50 steel exhibited a relatively fine and homogeneous ferrite grain distribution compared with C45 and 37MnB4 steels. For C50 steel, the mean ferrite grain area was 18.5 µm^2^ in the transverse section and 21.1 µm^2^ in the longitudinal section, while the mean maximum grain diameter was approximately 7.1–7.2 µm and the mean minimum grain diameter was approximately 4.0 µm. Approximately 49–50% of the analyzed ferrite grains had an area not exceeding 15 µm^2^. C50 steel was selected for the present investigation based on these results.

The C50 steel used in this study was supplied as hot-rolled bars with a nominal diameter of 60 mm in the normalized condition by Impakta Metal d.o.o. (Ljubljana, Slovenia). The CV joint inner races were subsequently manufactured from the normalized bars by machining prior to induction hardening.

The nominal chemical composition range of C50 steel according to EN 10083-2 is (wt.%): C 0.47–0.55; Mn 0.50–0.80; Si ≤ 0.40; P ≤ 0.025; S ≤ 0.035. The chemical composition was determined by spark optical emission spectroscopy (OES) using three independent specimens (n = 3). The values reported in Table 1 represent the arithmetic mean of the three measurements. Analyses were performed using a SPECTROMAXx LMX10 (SPECTRO Analytical Instruments GmbH, Kleve, Germany) optical emission spectrometer.

The initial microstructure consisted of ferrite and pearlite with a uniform phase distribution and no significant banding or segregation. As C50 is a hypoeutectoid steel, the approximate fractions of proeutectoid ferrite and pearlite can be estimated from the Fe–Fe_3_C equilibrium diagram using the lever rule [4,14].

The theoretical fractions of proeutectoid ferrite and pearlite were calculated using the lever rule as follows:

(1) $$f_{\alpha }=\frac{C_{E}-C_{0}}{C_{E}-C_{\alpha }}$$

(2) $$f_{P}=\frac{C_{0}-C_{\alpha }}{C_{E}-C_{\alpha }}$$

where *C*_0_ is the measured carbon content of the steel (0.50 wt.%), *C_E_* is the eutectoid carbon content (0.76 wt.%), and *C_α_* is the carbon solubility in ferrite at the eutectoid temperature (0.022 wt.%). Accordingly:

(3) $$f_{\alpha }=\frac{0.76-0.50}{0.76-0.022}=0.352\approx 35\%$$

(4) $$f_{P}=\frac{0.50-0.022}{0.76-0.022}=0.648\approx 65\%$$

**Figure 2:**
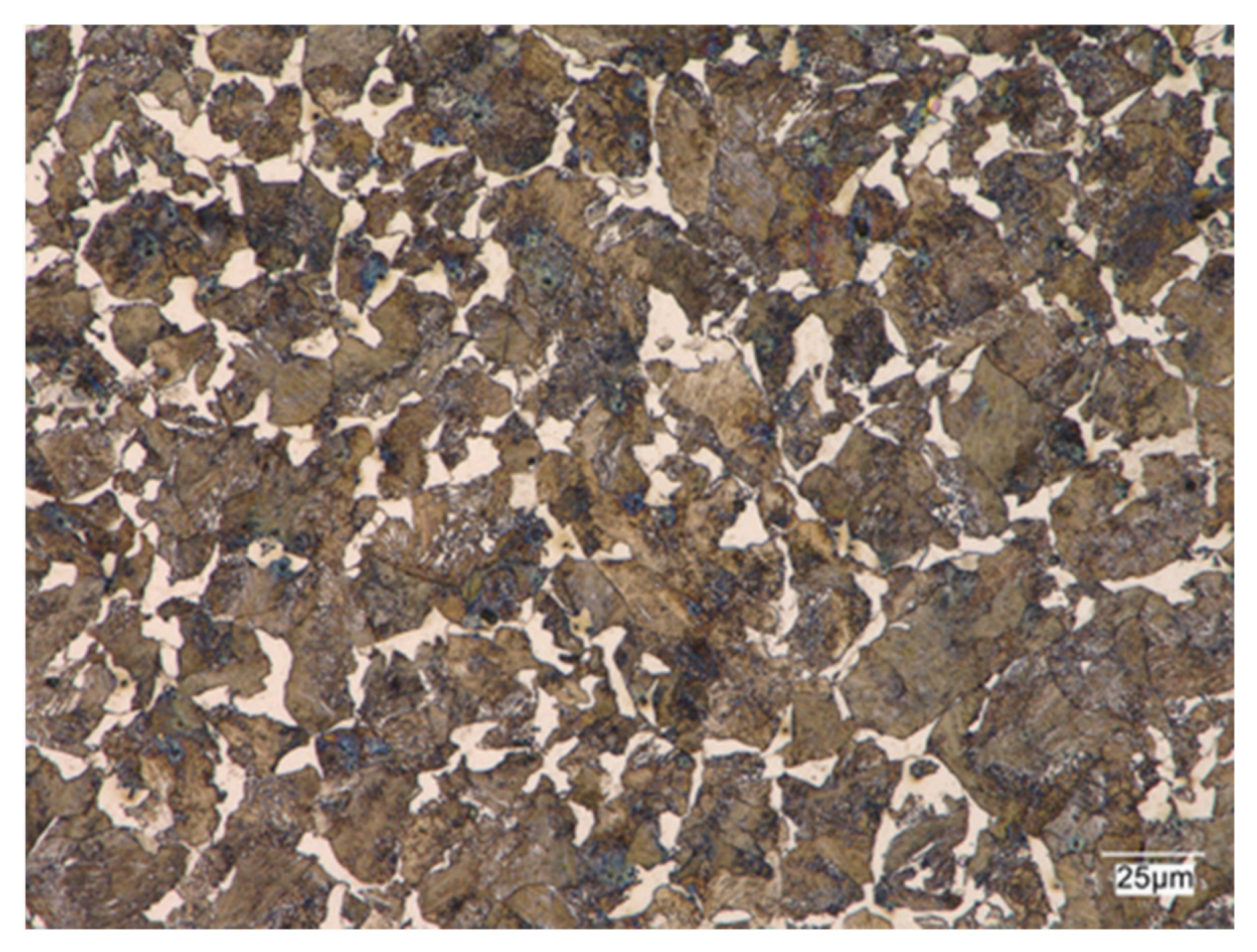
Representative optical micrograph of the investigated C50 steel in the normalized condition prior to induction hardening, showing the ferrite–pearlite microstructure; Nital etched.

These values represent theoretical equilibrium estimates rather than experimentally measured phase fractions. The normalized condition of the investigated material is consistent with the observed ferrite–pearlite microstructure (Figure 2). The initial microstructure was examined using an Axiovert 25 optical microscope (Carl Zeiss, Oberkochen, Germany).

The hardness in the delivery condition was measured using the Vickers hardness method in accordance with ISO 6507-1 [26], with a test force of 98.07 N (HV10). Five independent measurements were performed, resulting in an average hardness of 219 ± 5 HV10 (n = 5). This value is consistent with normalized C50 steel and indicates sufficient core hardness while maintaining adequate carbon content for martensitic transformation during subsequent surface induction hardening.

### 2.2. Induction Hardening Procedure

Induction hardening trials were conducted using an industrial vertical CNC-controlled induction hardening system manufactured by SAET S.p.A. (Leinì, Turin, Italy). The system was equipped with an IGBT-based power converter for induction hardening, with a maximum rated power of 400 kW and an operating frequency range of 5–30 kHz. All experiments were performed at a fixed operating frequency of 19 kHz. At austenitizing temperatures, the effective electromagnetic penetration depth at 19 kHz is approximately 2–3 mm in medium-carbon steels, which is comparable to the target hardening depth (2–4 mm). This enables efficient subsurface heating while limiting excessive bulk thermal diffusion. No post-hardening tempering or stress relief treatment was applied; all specimens were characterized in the as-induction-hardened and quenched conditions.

**Figure 3:**
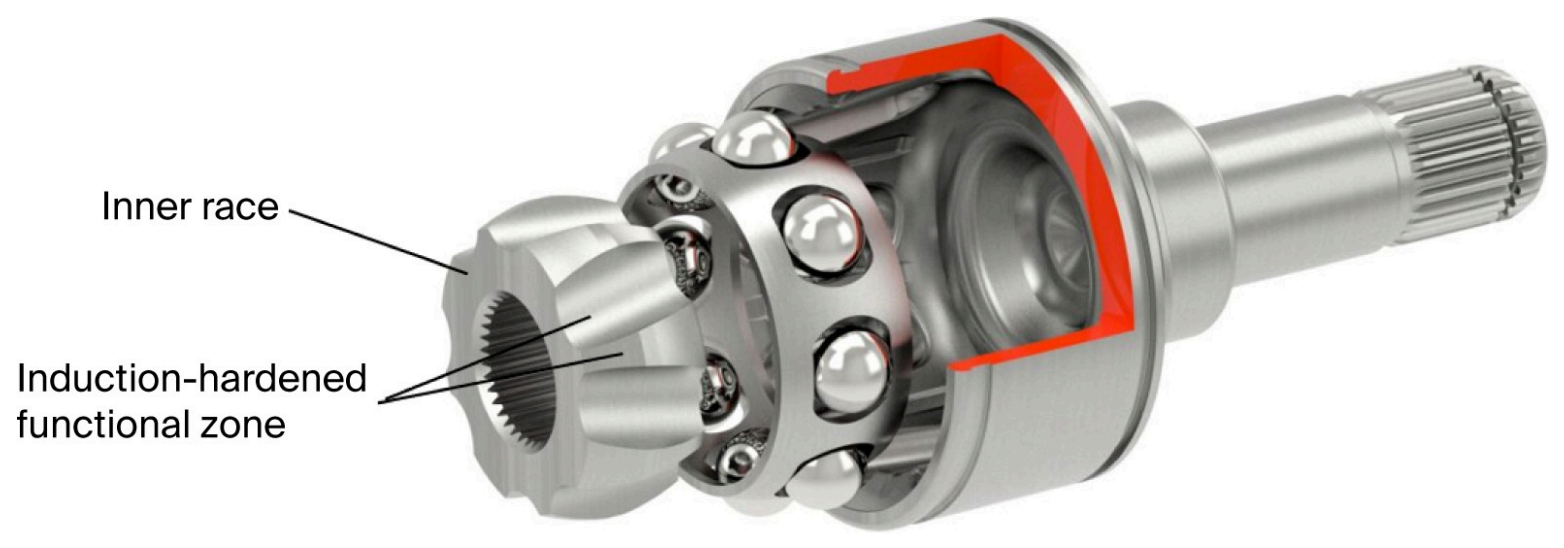
Schematic representation of a ball-type constant-velocity joint indicating the inner race and the functional zone subjected to induction hardening.

**Figure 4:**
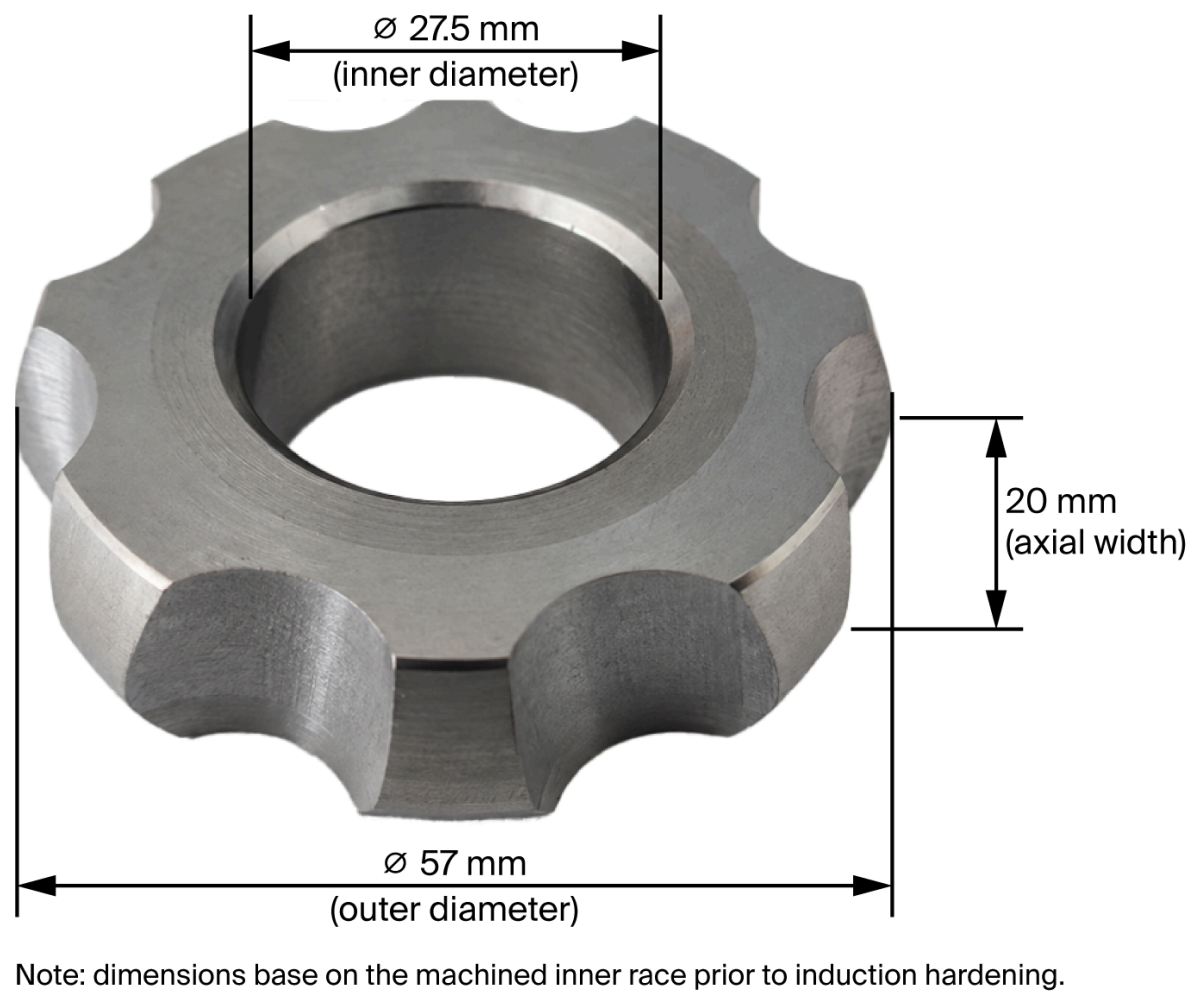
Geometry and principal dimensions of the C50 steel inner race prior to induction hardening.

The investigated component was a machined C50 steel inner race within a ball-type CV joint assembly (Figure 3). Its geometry and principal dimensions prior to induction hardening are shown in Figure 4. The component had an overall diameter of 57 mm, an axial width of 20 mm, and an internal diameter of 27.5 mm. These dimensions provide the geometrical context for the single-shot and scanning induction hardening configurations described below.

The functional hardening zone comprised the ball-track raceways and the inter-track outer diameter surface. A conformal copper coil with integrated water cooling channels was designed to follow the curvature of the raceway. During heating, the component was rotated at 150 rpm to ensure circumferential thermal uniformity.

Two induction hardening strategies were investigated: single-shot and scanning.

In the single-shot strategy, the entire functional raceway region was heated simultaneously for a defined heating time (t) of 28 s, with the coil remaining stationary in the axial direction. This results in high instantaneous power density and extended thermal exposure, promoting deeper thermal penetration.

**Figure 5:**
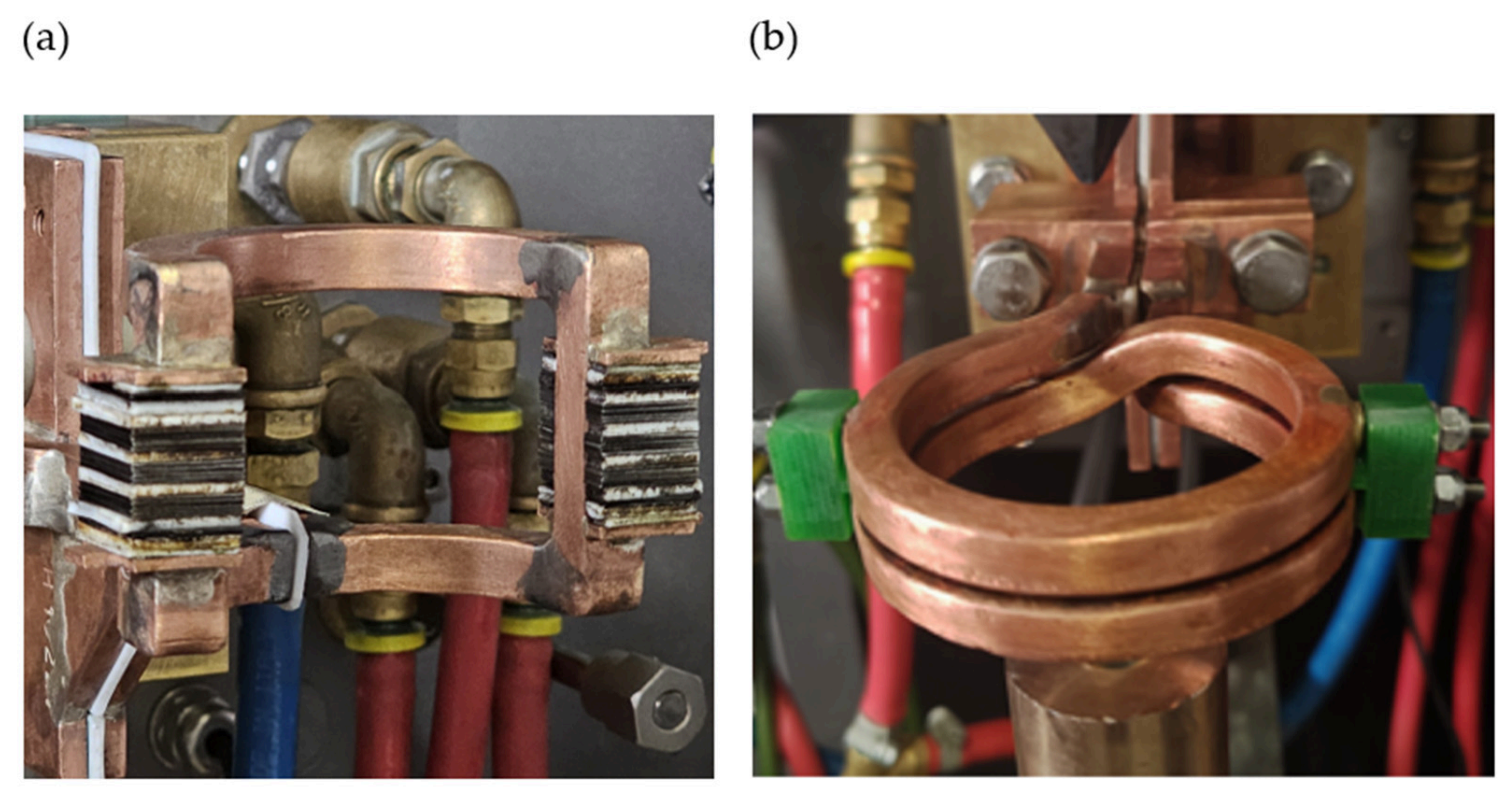
Induction coils used for the hardening experiments: (**a**) single-shot configuration and (**b**) scanning configuration.

The experimental setups used for the single-shot and scanning induction hardening strategies are shown in Figure 5. In the scanning strategy, the coil moved progressively along the axial direction of the raceway at a controlled feed rate (v) of 5 mm·s^−1^. Each surface element was therefore exposed to the electromagnetic field for a shorter duration compared with the single-shot configuration, resulting in localized heating and localized thermal gradients along the raceway. The induction coils used for both configurations had a nominal effective inner diameter of 61 mm. With the component outer diameter of 57 mm, this corresponded to a nominal radial coil-to-part lift-off distance of 2 mm.

The principal process parameters are summarized in Table 2.

To quantify thermal input, the following energy descriptors were defined:

*E*= *P*⋅ *t* (5)

(6) $$E_{\mathrm{lin}}=\frac{P}{v}$$

where *P* is generator power (kW), *t* is heating time (s), and *v* is axial feed rate (mm·s^−1^). These descriptors were used to correlate process parameters with hardness distribution, effective case depth (EDH_500_HV), and microstructural homogeneity. The percentage power values reported in Table 2 correspond to the power setpoints entered in the machine control system (HMI) and should not be interpreted as a direct percentage of the 400 kW maximum rated generator power. The corresponding generator power values indicated by the HMI during processing were approximately 50 kW for the single-shot condition and 66 kW for the scanning condition. Based on these values, the calculated energy-input descriptor for the single-shot condition was E = 50 × 28 = 1400 kJ, while the corresponding linear energy-input descriptor for the scanning condition was E_lin_ = 66/5 = 13.2 kJ·mm^−1^. The calculated values represent process-energy descriptors based on the HMI-indicated generator power and should not be interpreted as the total electrical energy consumption of the equipment.

One representative industrial processing condition was compared for each induction hardening strategy. The conditions were 50 kW with a heating time of 28 s for the single-shot strategy and 66 kW with a coil feed rate of 5 mm·s^−1^ for the scanning strategy. The objective of the study was to compare the resulting hardened-layer characteristics and tribological response of the two processing approaches rather than to establish a complete process parameter matrix. The investigated parameter window was selected to achieve effective case depths within the 2–4 mm range required for CV joint inner race applications.

Each of the two investigated processing conditions was repeated three times (n = 3). Table 2 therefore represents the complete experimental matrix used in the present comparative study.

### 2.3. Hardness and Microstructural Characterization

#### 2.3.1. Sample Preparation

After induction hardening, specimens were sectioned perpendicular to the raceway surface along the axial direction using an Accutom precision cutting machine (Struers ApS, Ballerup, Denmark) with continuous coolant supply to prevent tempering effects. Cross-sections were mounted in conductive resin, ground using SiC abrasive papers up to 1200 grit, and polished using diamond suspensions down to 1 µm.

#### 2.3.2. Hardness Measurement and Effective Case Depth Determination

Two separate Vickers hardness measurement series were performed. HV10 measurements were used exclusively to characterize the C50 steel in the delivery condition prior to induction hardening, as described in Section 2.1. After induction hardening, HV1 measurements were performed on metallographic cross-sections to determine the hardness profiles and effective case depth. These post-hardening measurements were carried out on three independent cross-sections (n = 3) for each processing condition.

Cross-sectional hardness profiles were recorded along a line normal to the treated surface using the Vickers method in accordance with ISO 6507-1 [26]. The first indentation was placed at a depth of 0.10 mm below the surface to avoid edge effects. Subsequent indentations were performed at constant depth increments of 0.20 mm across the hardened layer and transition zone and continued into the core until the hardness stabilized at the base material level. A minimum spacing of at least three times the indentation diagonal was maintained to prevent interaction with plastic deformation zones.

The effective case depth (EDH_500_HV) was defined in this study as the perpendicular distance from the treated surface to the depth at which the hardness decreased to 500 HV1. The hardness-depth measurement procedure follows ISO 18203:2026 [18] for surface-hardened layers. The 500 HV1 threshold was adopted in the present study as a functional criterion for determining the effective case depth of the investigated induction-hardened CV joint inner races.

When the hardness profile crossed the 500 HV1 level between two adjacent measurement points, linear interpolation was applied to determine the corresponding depth. For each process condition, three independent cross-sections (n = 3) were evaluated. At each measurement depth, hardness values were calculated as the arithmetic mean of the three measurements and are reported together with the corresponding standard deviation (SD). The effective case depth (EDH_500_HV) was determined independently for each hardness profile by linear interpolation between the two adjacent measurement points surrounding the 500 HV1 threshold. The resulting EDH_500_HV values are reported as mean ± standard deviation (n = 3).

**Figure 6:**
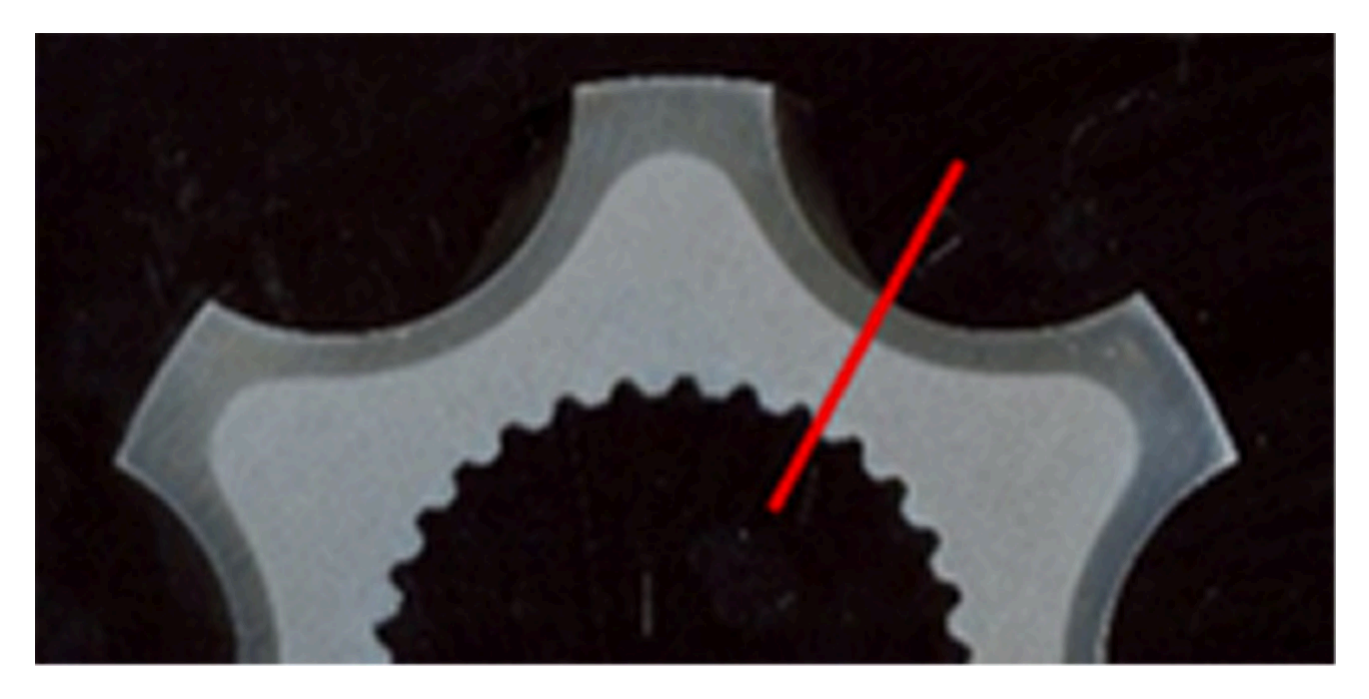
Representative macro cross-section of an induction-hardened inner race (scanning strategy). The red line indicates the measurement line used for cross-sectional hardness evaluation.

The location of hardness measurements is illustrated in Figure 6. Hardness profiles for both strategies were recorded along equivalent cross-sectional positions. Hardness profiles and effective case depth measurements were intentionally performed in the functionally critical ball–raceway contact region of the inner race. The objective was to characterize the hardened layer in the zone directly subjected to service contact loading rather than to quantify the circumferential variation in case depth over the entire component. For each processing condition, three independent cross-sections (n = 3) were evaluated at equivalent functional locations.

#### 2.3.3. Microstructural Examination

The microstructure was characterized in several stages. First, a macroscopic analysis of cross-sectional specimens was carried out to evaluate the distribution, continuity, and shape of the hardened layer after induction hardening. For microstructural observations, the metallographically prepared specimens were etched with Nital to reveal the microstructure.

Detailed microstructural observations were subsequently made using the same Axiovert 25 optical microscope (Carl Zeiss, Oberkochen, Germany) used for the initial microstructure characterization shown in Figure 2, and a JEOL 6610 scanning electron microscope (JEOL Ltd., Tokyo, Japan). SEM observations were carried out at an accelerating voltage of 20 kV and a working distance of approximately 10 mm using secondary electron imaging (SEI). Observations were conducted in an area located approximately 0.75 mm below the treated surface, corresponding to the subsurface region relevant to the maximum shear stresses under Hertzian contact conditions.

The microstructural assessment was qualitative. Optical and SEM images were used to compare the morphology and apparent homogeneity of the hardened microstructure and to identify local microstructural heterogeneities associated with the applied induction hardening strategy. No quantitative stereological or image analysis procedure was applied; therefore, we did not determine parameters such as retained austenite fraction, martensite lath or packet size, and the quantitative fraction of locally heterogeneous regions.

### 2.4. Tribological Testing

Tribological performance was evaluated for specimens treated using both induction hardening strategies (single-shot and scanning). Testing was conducted using a reciprocating ball-on-flat configuration under dry sliding conditions.

#### 2.4.1. Test Configuration and Parameters

**Figure 7:**
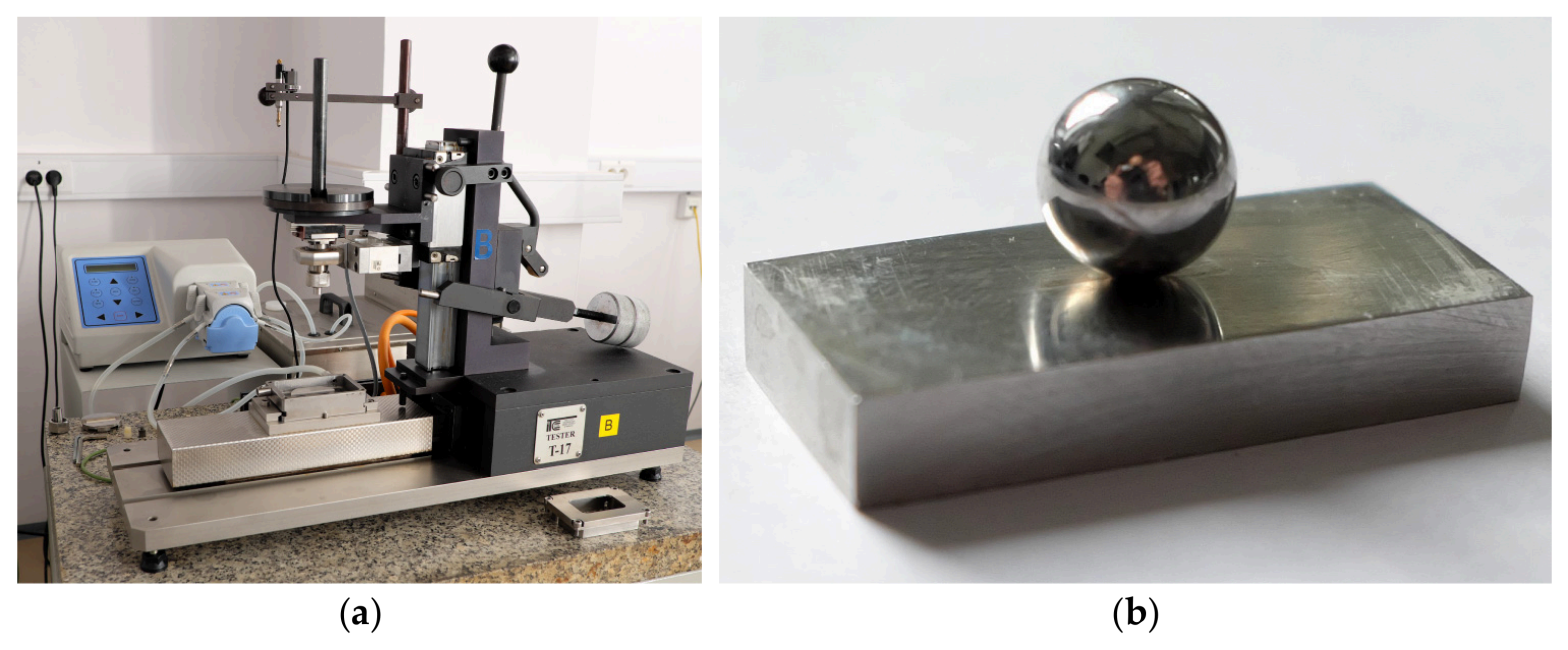
(**a**) T-17 tribometer used for reciprocating friction and wear testing; (**b**) ball-on-flat test configuration.

Experiments were performed using a T-17 reciprocating tribometer (ITeE-PIB, Radom, Poland) operating in linear sliding mode. The induction-hardened raceway surface served as the flat specimen. A 100Cr6 bearing steel ball (diameter 10 mm, hardness ≈ 62 HRC) was used as the counterbody. The selection of 100Cr6 was based on its widespread application in rolling contact systems and its stable mechanical behavior under dry sliding conditions. The tribological test setup and the ball-on-flat configuration used in this study are shown in Figure 7.

The principal test parameters are summarized in Table 3.

The total sliding distance *L* was calculated using

*L* = 4*sN* (7)

where

*s*—half-stroke length;

*N*—number of cycles.

One reciprocating cycle was defined as one complete forward-and-return motion. Based on a half-stroke amplitude of 2.5 mm and 28,800 complete reciprocating cycles, the total sliding distance was 288 m.

#### 2.4.2. Contact Pressure Estimation

The initial maximum Hertzian contact pressure was estimated using classical elastic contact theory for a sphere-on-flat configuration [2,3].

The contact radius *a* was calculated as

(8) $$a={\left(\frac{3\mathrm{FR}}{4E^{*}}\right)}^{1/3}$$

where:

*F*—applied normal load;

*R*—ball radius (5 mm);

E^*^—reduced elastic modulus of the contacting bodies.

The maximum contact pressure *p*_0_ was then determined as

(9) $$p_{0}=\frac{3F}{2\pi a^{2}}$$

The reduced elastic modulus E^*^ of the contacting bodies was calculated according to

(10) $$1/E^{*}=\left(1-\nu_{1}^{2}\right)/E_{1}+\left(1-\nu_{2}^{2}\right)/E_{2}$$

Assuming *E*_1_ = *E*_2_ = 210 GPa and *ν*_1_ = *ν*_2_ = 0.3 for steel–steel contact, the reduced elastic modulus is E^*^ ≈ 115.4 GPa. Using this value, the Hertzian contact radius was calculated as approximately 99 µm, resulting in an estimated maximum Hertzian contact pressure of approximately 1.46 GPa under the applied normal load of 30 N. This pressure level corresponds to subsurface stress magnitudes relevant to rolling–sliding contact conditions encountered in CV joint raceways.

Three independent tribological tests were performed for each induction hardening strategy (n = 3). Statistical comparisons between the scanning and single-shot groups were performed using a two-sided Welch’s independent-samples *t*-test, which does not assume equal variances. Statistical significance was evaluated at α = 0.05. Results are reported as mean ± standard deviation (SD).

## 3. Results

### 3.1. Hardness and Effective Case Depth

Both induction hardening strategies achieved near-surface hardness values within the target range of 653–772 HV1. The scanning-treated specimens exhibited near-surface hardness values of approximately 700–780 HV1, whereas the single-shot specimens showed values of approximately 670–740 HV1. However, the single-shot condition displayed a broader hardness dispersion within the subsurface region.

Although the average hardness levels in the near-surface region were comparable, the single-shot strategy exhibited greater variability across the evaluated cross-sections. The scanning process showed a more consistent hardness distribution and lower variability in effective case depth compared with the single-shot treatment.

**Figure 8:**
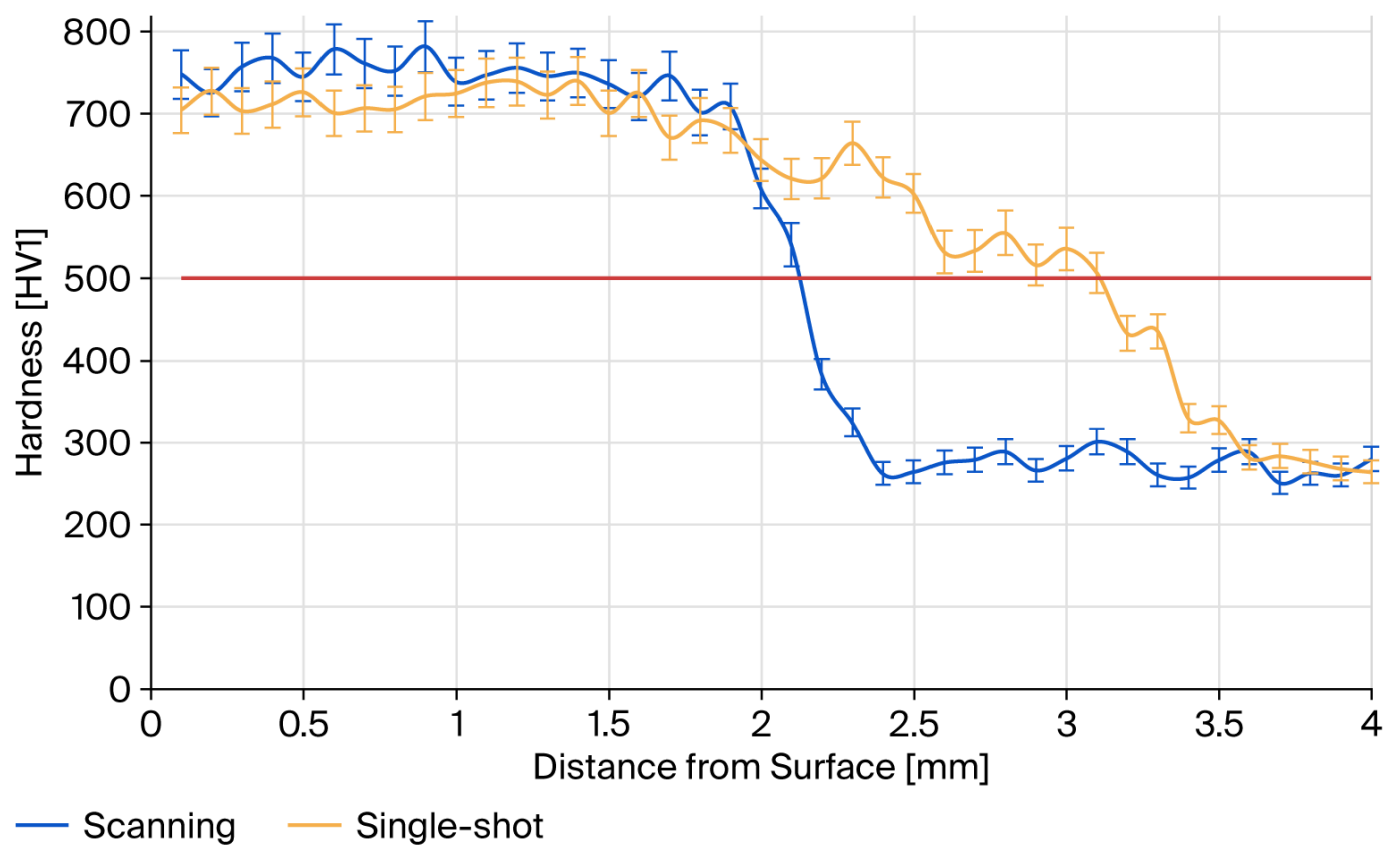
Cross-sectional hardness profiles measured normal to the raceway surface for scanning and single-shot induction hardening strategies. Data are presented as mean ± standard deviation (n = 3 independent cross-sections). The red line indicates the 500 HV1 criterion used to determine effective case depth (EDH_500_HV).

Cross-sectional hardness profiles (Figure 8) revealed distinct differences in effective case depth. The mean EDH_500_HV was 2.13 ± 0.02 mm for the scanning strategy and 3.04 ± 0.15 mm for the single-shot strategy (n = 3). Thus, the single-shot process produced a deeper hardened layer while also exhibiting greater variability in effective case depth.

The greater thermal penetration observed in the single-shot strategy is associated with simultaneous exposure of the entire functional zone to high electromagnetic power density, resulting in prolonged austenitization and enhanced subsurface heat diffusion [8,21]. In contrast, the scanning approach introduces controlled linear energy input and shorter local thermal exposure, limiting excessive bulk heating and enabling improved case depth control.

From a functional standpoint, both strategies satisfied the minimum near-surface hardness requirement. However, the scanning method demonstrated lower variability in hardness distribution within the subsurface region most relevant to rolling–sliding contact conditions in CV joint applications.

The core hardness values obtained from the HV1 profiles were slightly higher than the initial bulk hardness of 219 ± 5 HV10. This difference is attributed mainly to the different indentation loads. HV1 measurements sample a smaller material volume and are therefore more sensitive to local variations within the ferrite–pearlite microstructure, whereas HV10 measurements provide a more spatially averaged hardness value over a larger indentation area. Consequently, the HV1 and HV10 values should not be considered directly equivalent.

### 3.2. Microstructural Characterization

**Figure 9:**
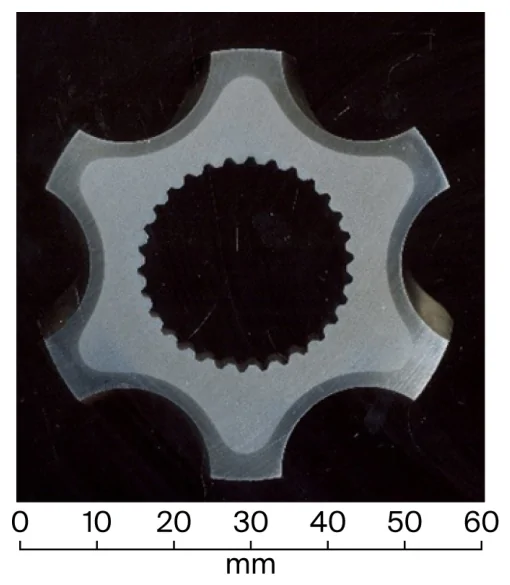
Macroscopic image of the cross-section of the component after induction hardening using the scanning method.

**Figure 10:**
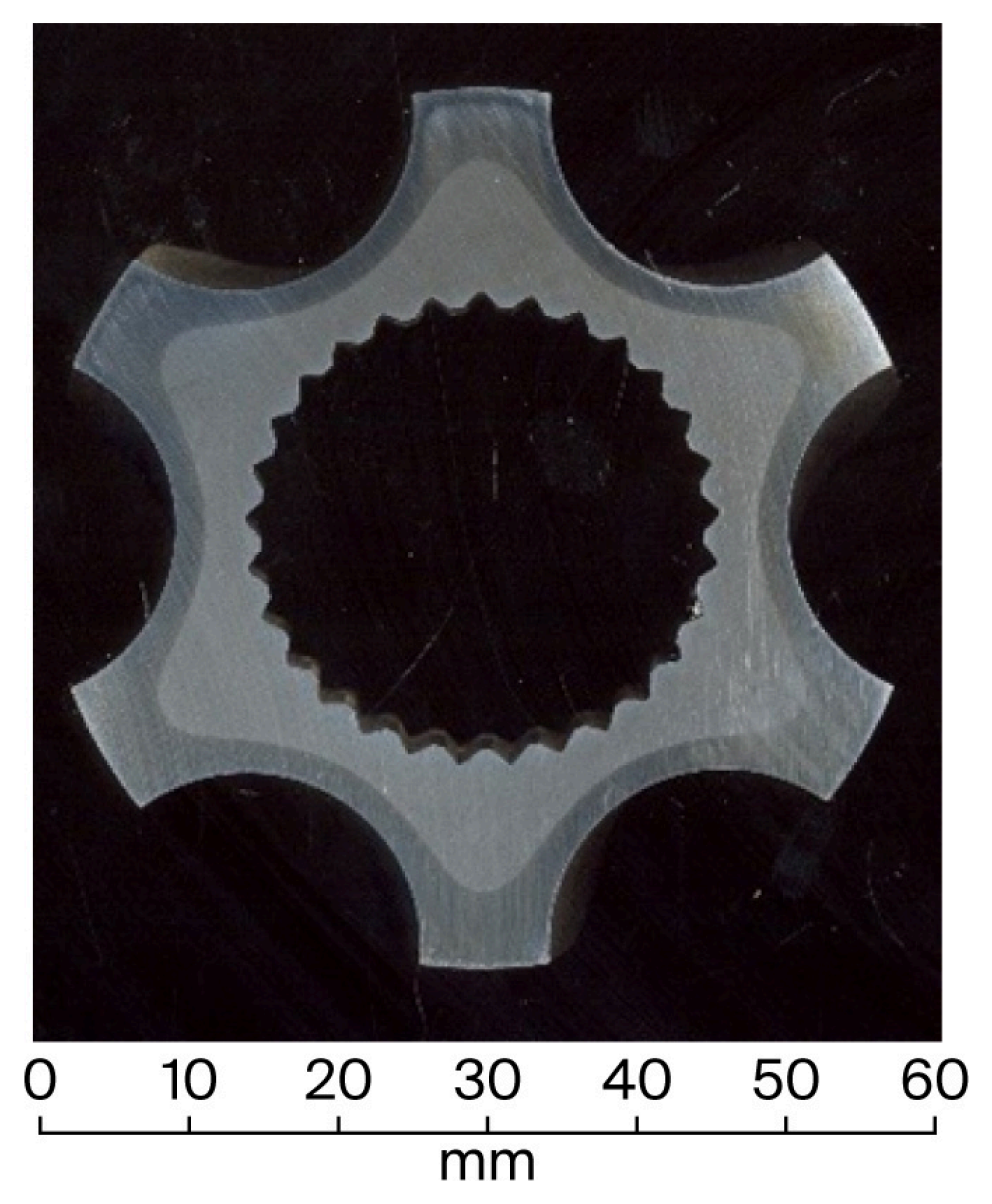
Macroscopic image of the cross-section of the component after induction hardening using the single-shot method.

In the first stage of the study, a macroscopic analysis of cross-sectional specimens was carried out to evaluate the distribution and continuity of the hardened layer after induction hardening. Images of the macrostructures are presented in Figure 9 and Figure 10.

Based on the macrostructural observations, a clearly defined and continuous hardened layer was obtained for both induction hardening strategies. Within the analyzed cross-sections corresponding to the functionally critical raceway region, the scanning-treated specimens exhibited a visually more regular hardened-zone boundary, whereas those treated with the single-shot method showed greater local variation in layer geometry, including locally increased case thickness at the component peaks. In the single-shot condition, the observed microstructure also exhibited predominantly martensitic morphology; however, the lath-like features were less clearly defined and the examined fields contained local regions showing different optical contrast and morphology from the surrounding matrix. These observations indicate greater local morphological heterogeneity within the analyzed fields of view.

It should be emphasized that this comparison represents a qualitative assessment of the analyzed cross-sections and does not constitute a quantitative evaluation of circumferential case-depth uniformity over the entire component. Therefore, these observations should be interpreted as indicating local differences in hardened-layer geometry rather than as a quantitative measure of overall circumferential uniformity or process repeatability.

**Figure 11:**
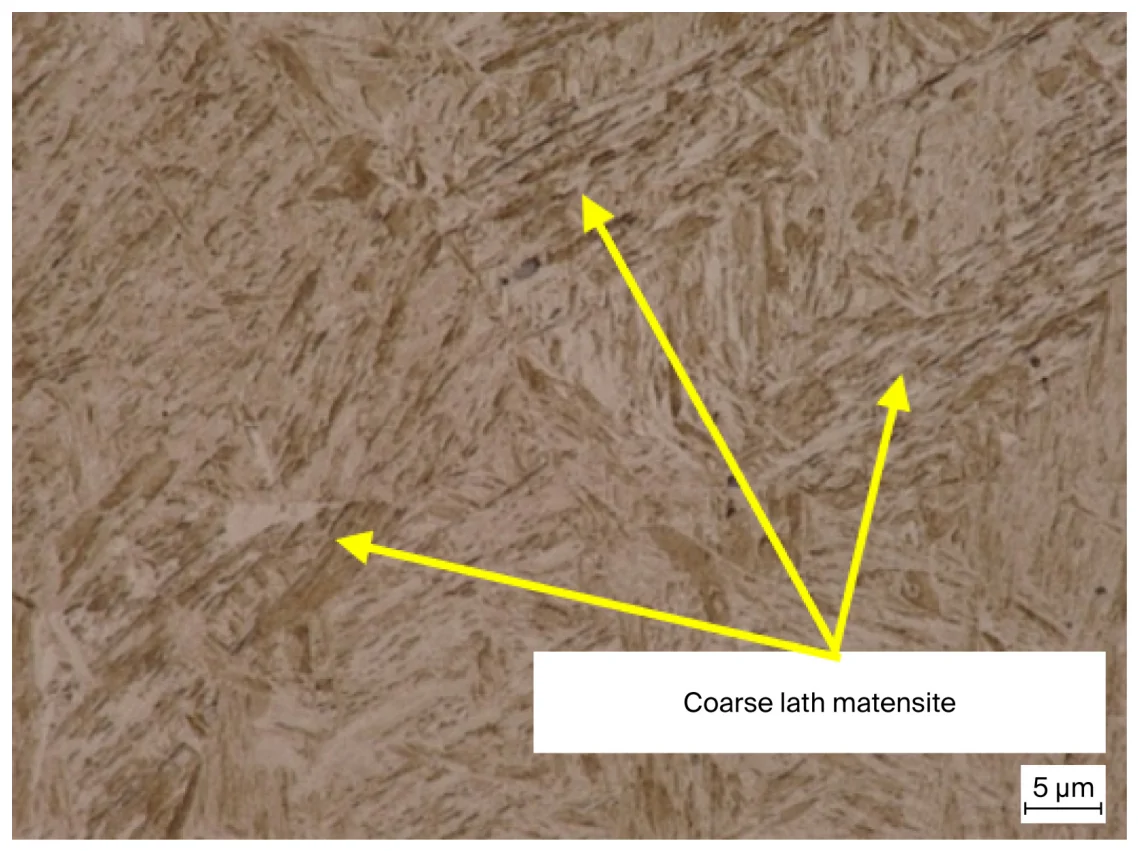
Microstructure of the hardened layer after scanning induction hardening, observed by optical microscopy at 0.75 mm below the surface. The arrows indicate regions of coarse lath martensite. Scale bar: 5 µm. Nital-etched specimen.

**Figure 12:**
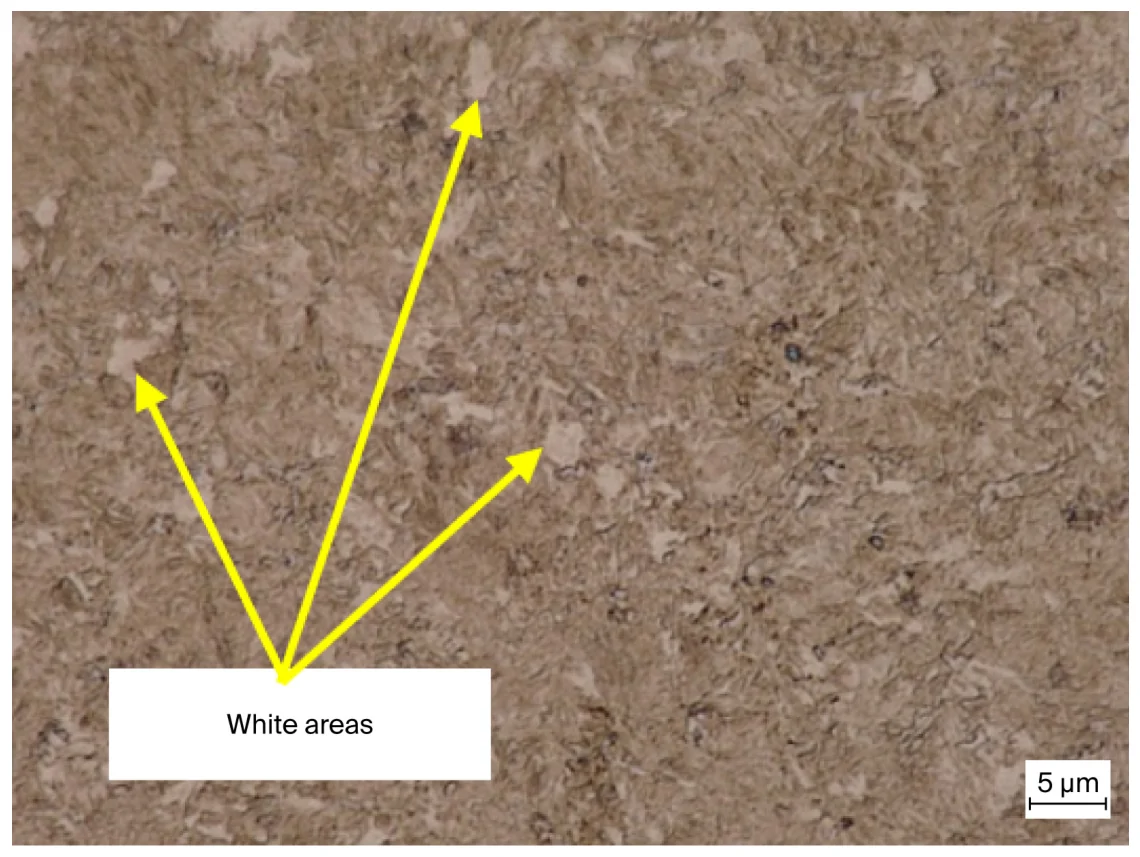
Microstructure of the hardened layer after induction hardening using the single-shot method, observed by optical microscopy at 0.75 mm below the surface. The arrows indicate local bright areas differing in contrast and morphology from the surrounding martensitic matrix. Scale bar: 5 µm. Nital-etched specimen.

A detailed microstructural analysis was carried out using optical microscopy for both induction hardening variants. Observations were conducted in an area located approximately 0.75 mm below the surface, corresponding to the region of maximum subsurface shear stress under Hertzian contact conditions and therefore relevant to the service performance of the raceway. Microstructural images obtained for both variants are presented in Figure 11 and Figure 12.

The microstructural images obtained for the single-shot and scanning methods indicate a predominantly martensitic structure; however, clear differences resulting from the applied induction hardening method are visible. In the case of hardening by the scanning method, a distinctly developed, relatively coarse lath martensitic structure is observed, visible in the form of well-recognizable martensite lath packets (Figure 11). This structure is characterized by high homogeneity throughout the analyzed area, with no visible remnants of phases present before induction hardening. The observed homogeneous martensitic microstructure is consistent with sufficiently uniform austenitization followed by martensitic transformation during quenching [10,14,15]. The continuity of the hardened layer was additionally confirmed by the corresponding macrostructural observations and hardness profiles.

In the case of the single-shot method, a martensitic microstructure was also obtained; however, it showed a significantly higher degree of refinement and dispersion. The images do not reveal martensite lath packets as clearly as in the sample hardened by the scanning method. In addition, bright areas are visible in the structure, clearly differing from the surrounding martensitic matrix (Figure 12). These regions indicate local microstructural heterogeneity within the analyzed area. However, their phase constitution cannot be identified unambiguously through optical microscopy alone; therefore, they were not assigned to a specific phase or transformation product in the present study.

**Figure 13:**
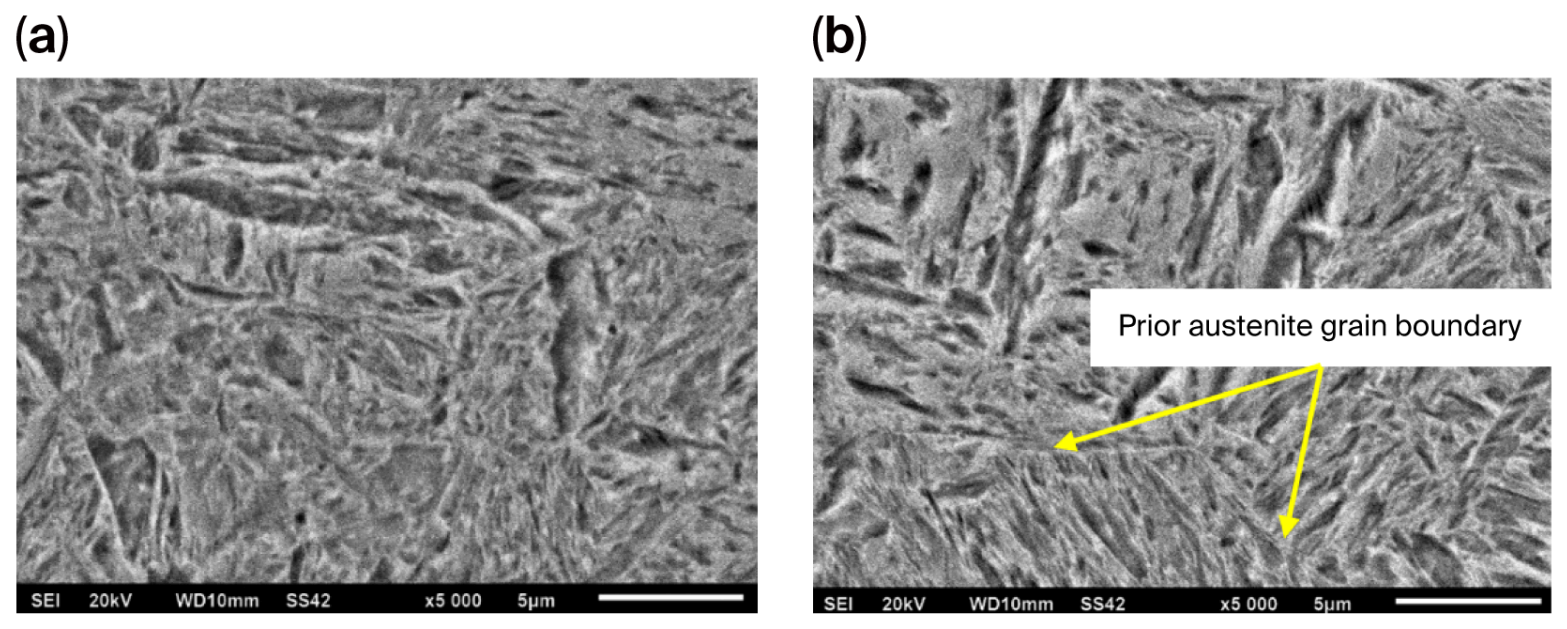
Microstructure of the hardened layer after induction hardening using the scanning method, observed by means of scanning electron microscopy at 0.75 mm below the surface. The arrows indicate locally visible prior austenite grain boundaries: (**a**) magnification 3000×, (**b**) magnification 5000×.

**Figure 14:**
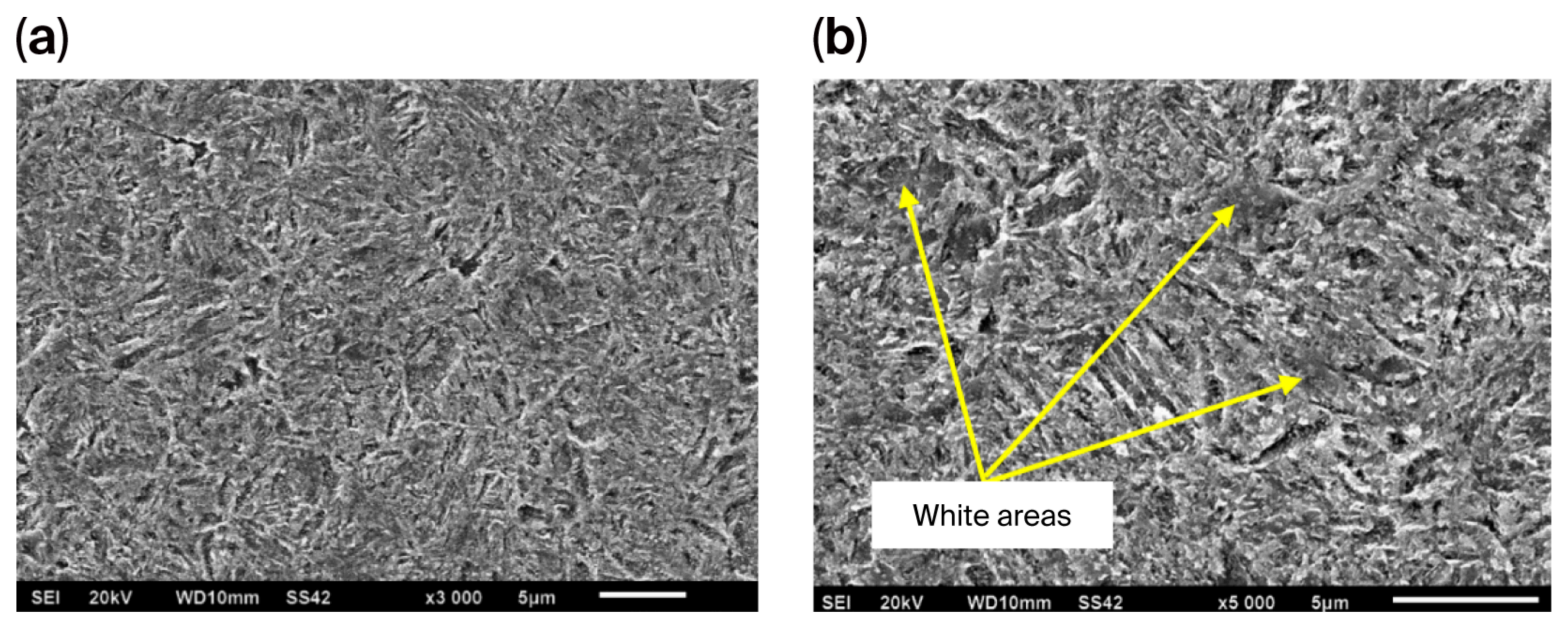
Microstructure of the hardened layer after induction hardening using the single-shot method, observed by means of scanning electron microscopy at 0.75 mm below the surface. The arrows indicate local regions differing in contrast and morphology from the surrounding martensitic matrix: (**a**) magnification 3000×, (**b**) magnification 5000×.

To gain a deeper understanding of the obtained microstructure, additional investigations were carried out using scanning electron microscopy. Microstructural images for scanning and single-shot induction hardening methods are presented in Figure 13 and Figure 14.

In the case of the single-shot method, a martensitic structure was also obtained, but it was characterized by a much greater degree of dispersion and lower homogeneity. SEM observations of the single-shot condition revealed local regions differing in contrast and morphology from the surrounding predominantly martensitic matrix. These observations confirm the presence of local microstructural heterogeneity; however, the phase constitution of these regions cannot be determined unambiguously from conventional SEM morphology alone. At the same time, martensite lath packets were not as clearly defined as in the case of the scanning strategy. The observed microstructure indicates a less favorable thermal cycle, leading to the formation of local structural heterogeneities, as reported for induction-hardened medium-carbon steels under non-uniform heating conditions [13,14]. This observation is consistent with the hardness results, which for the single-shot strategy exhibited lower values and increased scatter, ranging from approximately 670 to 740 HV1.

Although the single-shot method enabled a greater mean effective case depth (EDH_500_HV) of 3.04 ± 0.15 mm, the microstructural quality and homogeneity of the near-surface layer were inferior compared with those obtained using the scanning strategy.

From the service point of view, a homogeneous, fully martensitic, and stable structure in the zone corresponding to the occurrence of maximum subsurface shear stresses is more desirable. In this respect, the scanning method proved to be more advantageous, as it provided greater microstructural homogeneity, higher hardness in the near-surface region, and lower variability under the investigated conditions. Although the tribological tests did not reveal a clear superiority of either method regarding friction coefficient and wear, the microstructural and hardness results indicate that the scanning method better meets the requirements imposed on the surface layer of raceway elements operating under contact loading conditions. Consequently, it can be concluded that among the analyzed induction hardening variants, the scanning method was the more favorable solution.

### 3.3. Tribological Performance

The average steady-state coefficient of friction (COF) was comparable for both induction hardening strategies. As summarized in Table 4, the scanning-treated specimens exhibited a mean friction coefficient of approximately μ ≈ 0.49, whereas the single-shot specimens showed μ ≈ 0.48. The observed differences fall within the experimental scatter and do not indicate statistically significant variation in steady-state friction behavior.

Mass loss measurements for the hardened specimens and the 100Cr6 counterbody also revealed comparable overall wear levels between the two strategies. As summarized in Table 5, both induction treatments resulted in identical average specimen mass loss (0.8 mg) and similar counterbody mass loss (~0.5 mg), confirming comparable global material removal under the applied test conditions.

Statistical analysis using a two-sided Welch’s *t*-test showed no statistically significant difference in the average coefficient of friction between the scanning and single-shot strategies (0.49 ± 0.02 and 0.48 ± 0.01, respectively; n = 3 per group; t = 0.775, df = 2.94, *p* = 0.496). Similarly, no statistically significant differences were detected for specimen mass loss (*p* = 1.000) or ball mass loss (*p* = 1.000). Given the limited sample size (n = 3 per group), these results indicate that no statistically significant differences were detected under the applied test conditions and should not be interpreted as evidence of statistical equivalence between the two hardening strategies.

**Figure 15:**
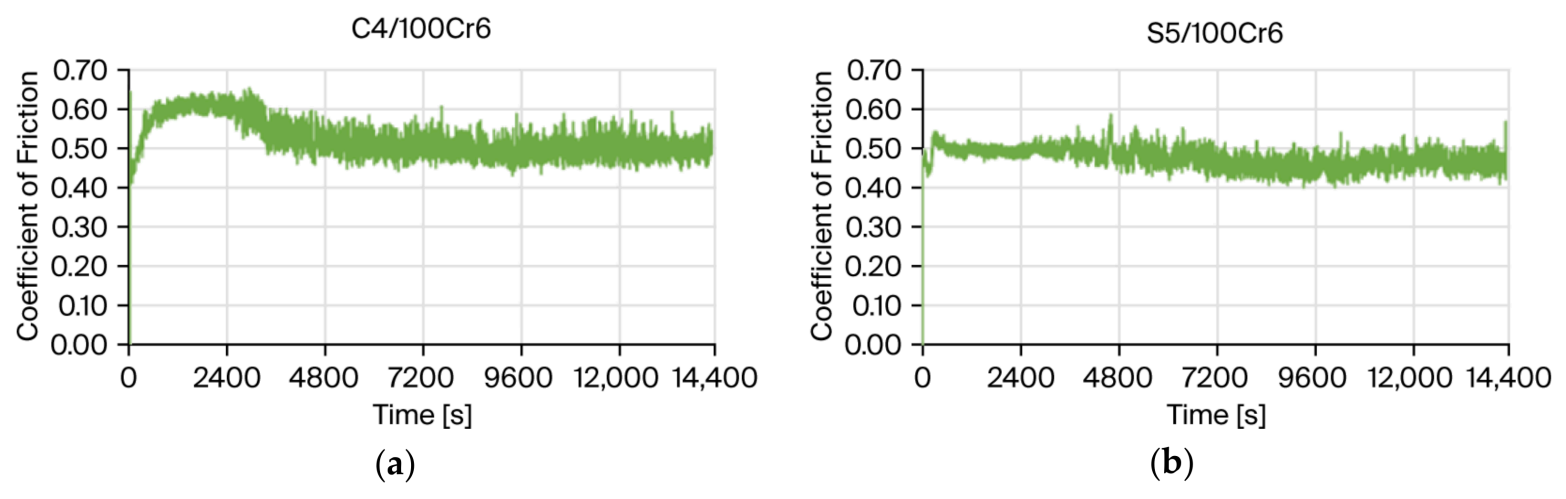
Evolution of the coefficient of friction as a function of sliding time for C50/100Cr6 material pairings under dry reciprocating conditions: (**a**) scanning-treated specimen; (**b**) single-shot-treated specimen. The first 3500 s correspond to the running-in stage.

Representative friction curves for both material pairings are presented in Figure 15. The initial stage of sliding, corresponding to the first 3500 s of testing, is considered the running-in period, and is defined based on stabilization of the coefficient of friction. During this phase, the friction coefficient increases and gradually stabilizes as the contacting surfaces adapt through initial asperity deformation and material transfer. After the running-in stage, both systems enter a steady-state regime characterized by relatively stable friction values with small-amplitude fluctuations. No abrupt instabilities or severe friction spikes were observed, indicating stable sliding conditions throughout the test duration.

**Figure 16:**
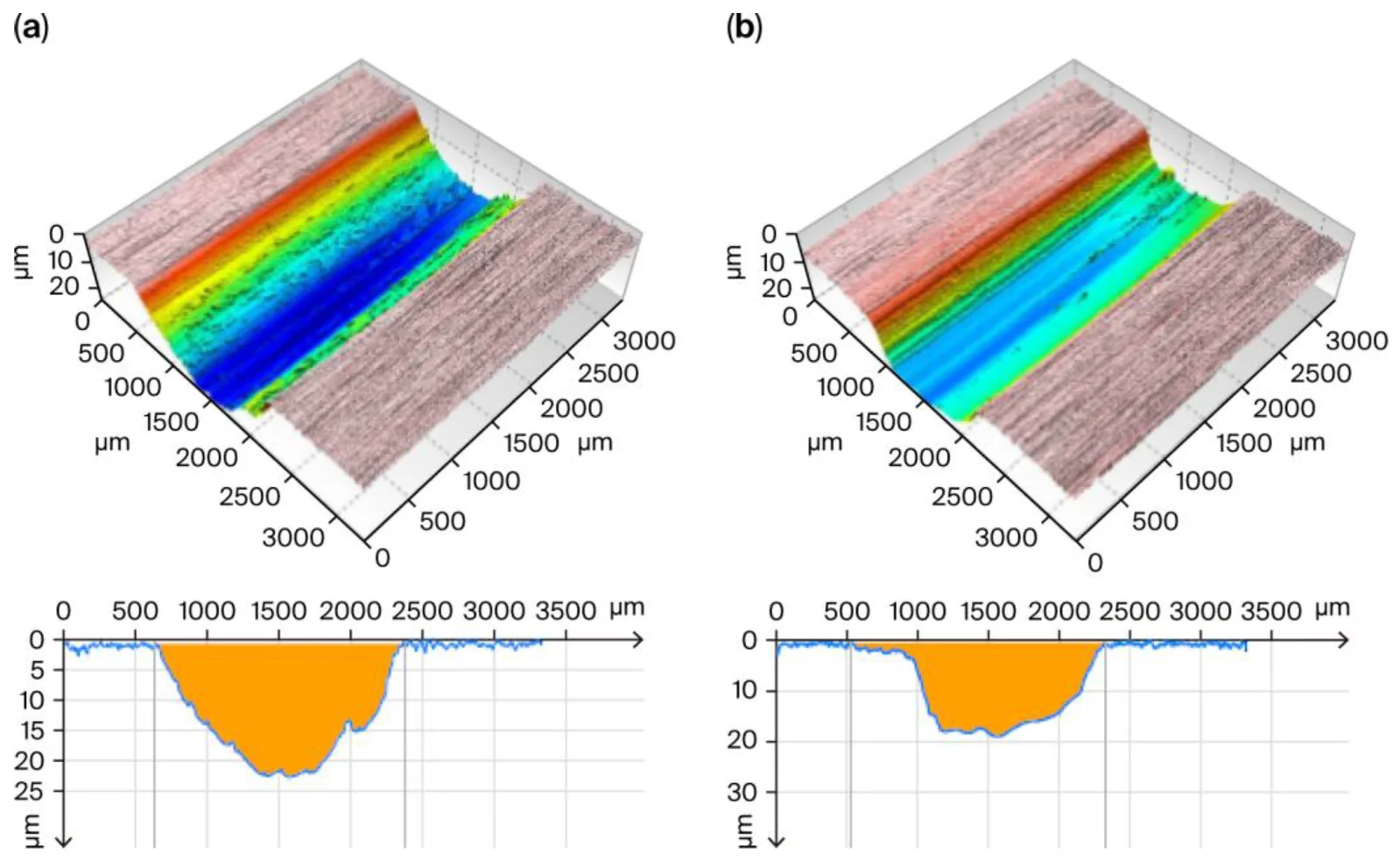
Representative 3D surface topographies and corresponding central cross-sectional profiles of wear tracks formed on the hardened specimens after reciprocating sliding tests: (**a**) scanning induction hardening and (**b**) single-shot induction hardening. The colors in the 3D maps represent relative surface height, with warmer colors indicating higher regions and cooler colors indicating lower regions.

**Figure 17:**
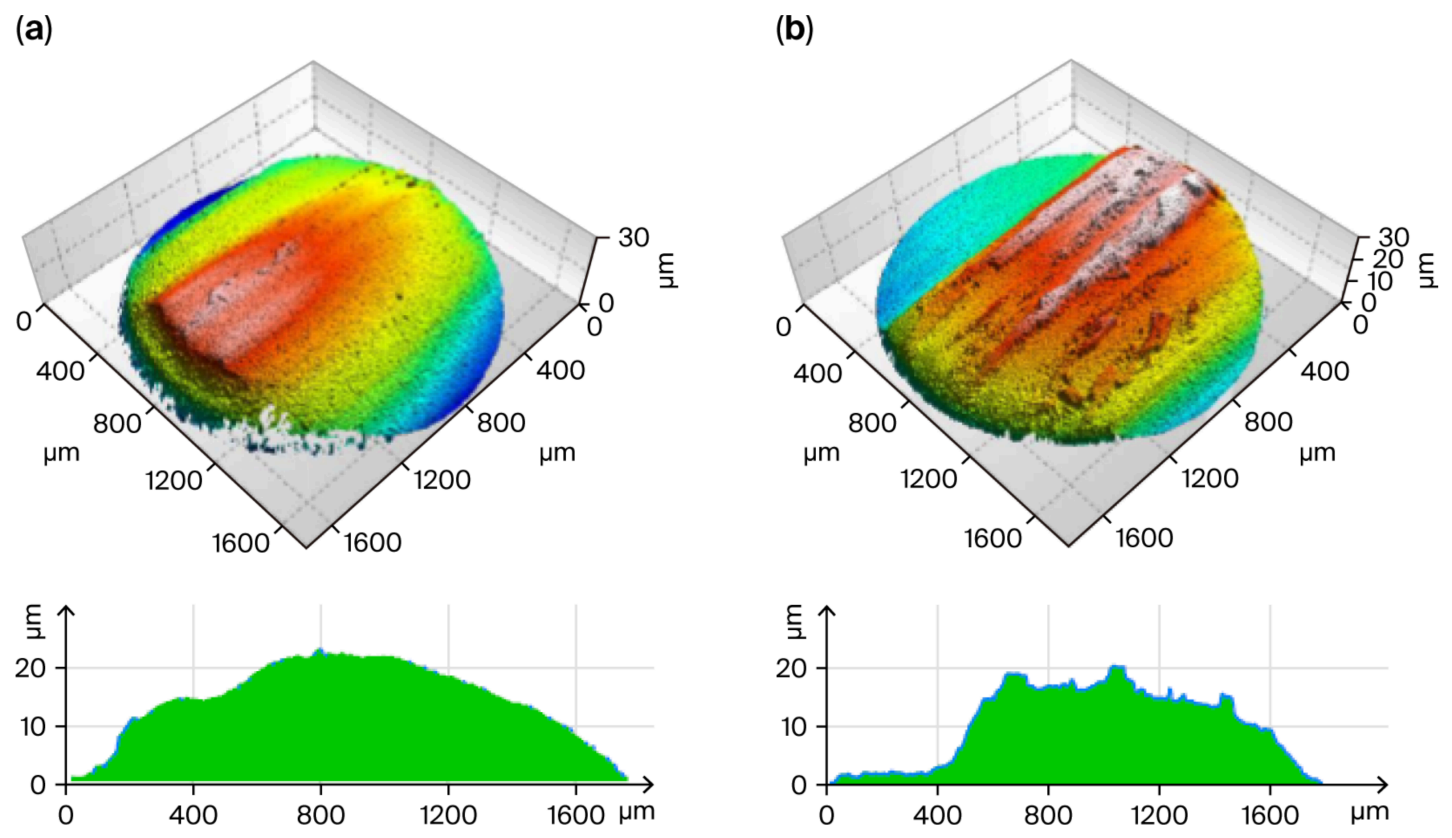
Representative 3D surface topographies and corresponding central cross-sectional profiles of wear scars formed on the 100Cr6 ball counterbodies after reciprocating sliding tests against the hardened specimens: (**a**) scanning induction hardening and (**b**) single-shot induction hardening. The colors in the 3D maps represent relative surface height, with warmer colors indicating higher regions and cooler colors indicating lower regions.

To further characterize wear behavior, geometrical parameters of the wear tracks were analyzed. The wear tracks exhibit typical morphology associated with mild adhesive–abrasive sliding, characterized by relatively smooth wear scars and limited surface damage. Representative surface topographies and central cross-sectional profiles of the wear tracks formed on the specimens are shown in Figure 16, while the corresponding wear scars formed on the 100Cr6 counterbody are presented in Figure 17.

The averaged measurements of wear tracks on specimens are summarized in Table 6, while the corresponding wear scar measurements on the balls are provided in Table 7.

Minor differences in wear scar geometry were observed between the two induction strategies. The single-shot specimens exhibited a slightly smaller average cross-sectional area of the wear track on the specimen surface, whereas the scanning-treated condition showed a marginally smaller cross-sectional area on the counterbody. These differences remained within the observed experimental scatter.

The relatively shallow wear depths, stable friction response, and absence of severe fluctuations indicate mild wear under stable contact conditions. The observed tendencies may be associated with differences in subsurface hardness gradients and microstructural continuity resulting from the respective induction strategies. Statistical analysis did not detect significant differences between the two hardening strategies in coefficient of friction or mass loss (*p* > 0.05). However, considering the limited number of independent tests (n = 3 per group), these findings should not be interpreted as demonstrating statistical equivalence.

Overall, both induction hardening strategies exhibited comparable friction behavior and wear levels under the investigated test conditions, with no statistically significant differences detected in coefficient of friction or mass loss.

### 3.4. Wear Mechanisms

The wear-track morphology and the relatively shallow wear depths observed for both hardening strategies indicate a mild mixed adhesive–abrasive wear mechanism under the applied dry reciprocating conditions. The continuous wear tracks and smooth cross-sectional profiles suggest that material removal occurred primarily through repeated micro-ploughing and local plastic deformation of surface asperities, accompanied by limited adhesive interaction between the hardened C50 surface and the 100Cr6 counterbody. During the running-in stage, asperity deformation and local material transfer are expected to contribute to the stabilization of the friction coefficient.

The wear response was similar for both hardening strategies despite differences in near-surface hardness and effective case depth. The scanning-treated specimens exhibited higher and more stable near-surface hardness, whereas the single-shot condition produced a deeper hardened layer. However, the comparable mass loss, wear-track dimensions, and friction coefficients indicate that these differences did not result in a clearly different dominant wear mechanism under the present test conditions. This is consistent with observations reported for heat-treated steels under dry reciprocating sliding, where wear behavior is governed by the combined effects of microstructure, hardness, surface deformation, and adhesive–abrasive interaction [27].

Local formation and release of wear debris may also contribute to third-body abrasion during reciprocating sliding. However, because no dedicated SEM/EDS analysis of the worn surfaces or collected debris was performed in the present study, the presence, composition, and specific role of transferred material or wear debris cannot be confirmed quantitatively. Therefore, the proposed wear-mechanism interpretation should be regarded as being based on the observed wear-track morphology, friction behavior, and measured wear parameters.

## 4. Discussion

The results indicate that the different induction hardening strategies had a significant effect on the morphology and homogeneity of the hardened layer, as well as on the hardness distribution and effective case depth. Although both analyzed variants produced a surface layer with a predominantly martensitic structure and hardness exceeding 650 HV1, clear differences were found between the scanning and single-shot methods regarding the continuity of the hardened zone, the stability of the hardness profiles, and the degree of microstructural homogeneity. In particular, the scanning method resulted in a more consistent hardened-layer geometry within the evaluated cross-sections and a more homogeneous near-surface microstructure, whereas the single-shot method promoted deeper thermal penetration at the expense of greater local structural heterogeneity.

Samples hardened by the scanning method achieved higher and more stable hardness in the near-surface region, at approximately 700–780 HV1, whereas for the single-shot variant the values were about 670–740 HV1. At the same time, a greater scatter of hardness values was observed in the subsurface region for the single-shot method, which may indicate lower stability of phase transformation conditions during heating and cooling. This observation is consistent with the metallographic results, in which the scanning variant exhibited a more homogeneous martensitic morphology, while the single-shot variant revealed local heterogeneities visible as bright areas in optical microscopy and as regions of different morphology in SEM observations. Such regions indicate local differences in microstructural morphology; however, their phase constitution cannot be determined unambiguously from the present OM and SEM observations.

A significant difference between the two hardening strategies concerns the effective case depth. The single-shot process led to the formation of a deeper hardened layer, with a mean EDH_500_HV of 3.04 ± 0.15 mm, whereas the scanning strategy resulted in a shallower effective case depth of 2.13 ± 0.02 mm, with lower variability than the single-shot strategy. This behavior results directly from the different modes of heat input. In the single-shot configuration, the entire functional zone is subjected simultaneously to a high electromagnetic power density for a relatively long time, which promotes deeper thermal penetration. In contrast, in the scanning method, each local surface segment is heated progressively and for a shorter time, which leads to a more localized thermal cycle and a sharper hardness transition toward the ferrite–pearlite core. This type of progressive heating provides improved control of thermal input and promotes more uniform phase transformation, as reported in previous studies on induction hardening of medium-carbon steels [8,11,12,13,21].

Regarding components operating under contact loading conditions, such as CV joint raceways, greater case depth is not the only evaluation criterion. The key issue is to obtain a hardened layer that sufficiently covers the subsurface stress field while maintaining high structural homogeneity in the region of maximum shear stress. In the present study, the microstructural observations were intentionally focused on the region located 0.75 mm below the surface, corresponding to the location of the maximum subsurface shear stresses under Hertzian contact conditions. In this zone, the scanning method provided a more homogeneous martensitic structure, with clearly developed martensite laths and locally visible prior austenite grain boundaries. In contrast, the single-shot variant showed a more dispersed martensitic morphology and the presence of regions locally deviating from the typical martensitic matrix, suggesting that despite greater effective case depth (EDH_500_HV), the quality of the microstructure in the most critical functional zone was less favorable.

This interpretation is also supported by the macrostructural observations. In both cases, a continuous hardened layer was obtained, confirming that both induction strategies are capable of effectively hardening the working zone of the component. Within the analyzed service-critical cross-sections, the scanning process exhibited a visually more regular hardened-zone boundary than the single-shot process. Because circumferential case-depth mapping was not performed, these observations should not be interpreted as a quantitative measure of full-component uniformity or process repeatability. In the single-shot variant, the hardened layer was also continuous, but its geometry was less regular and showed greater variation, including locally greater layer thickness at the peaks of the component. From a technological and industrial point of view, this is a less robust solution from a process stability perspective, since it may increase the sensitivity of the process to component geometry and to local overheating.

The tribological test results showed comparable friction behavior and similar material loss for both analyzed hardening variants under the applied dry reciprocating sliding conditions. Differences in wear track geometry remained within the limits of experimental scatter, and no clear superiority of either method was demonstrated in this simplified test configuration. This means that, within the investigated parameter range, the tribological response was determined primarily by the presence of a sufficiently hard martensitic surface layer rather than by moderate differences in the effective case depth. At the same time, the lack of a clear tribological advantage for the deeper single-shot variant indicates that increasing effective case depth alone does not automatically lead to improved service properties if the near-surface microstructure exhibits lower homogeneity.

A direct comparison of the hardened-layer characteristics with the tribological results indicates that the substantially greater effective case depth obtained for the single-shot condition did not result in a measurable improvement in friction or wear resistance under the applied test conditions. The single-shot strategy produced an EDH_500_HV of approximately 3.0–3.2 mm, compared with 2.0–2.2 mm for the scanning strategy, whereas the mean steady-state coefficients of friction were nearly identical (0.48 ± 0.01 and 0.49 ± 0.02, respectively). The mean specimen mass loss was 0.8 ± 0.1 mg for both processing conditions.

Only minor differences were observed in wear track geometry. The average wear track width was 1.66 ± 0.06 mm for the scanning condition and 1.70 ± 0.07 mm for the single-shot condition, while the maximum wear track depths were 20.4 ± 2.4 µm and 19.7 ± 3.1 µm, respectively. The corresponding cross-sectional wear track areas were 22,533 ± 1692 µm^2^ and 20,834 ± 3183 µm^2^. These differences remain within the experimental scatter and do not indicate a monotonic relationship between effective case depth and tribological performance.

The scanning-treated condition exhibited higher and more stable near-surface hardness and qualitatively more uniform microstructural morphology, whereas the single-shot condition produced a deeper hardened layer. However, neither of these differences resulted in a statistically conclusive tribological advantage under the present dry reciprocating sliding conditions. This suggests that, once a sufficiently hard martensitic surface layer is obtained, moderate differences in EDH_500_HV and qualitative microstructural morphology may have a limited influence on friction and mild wear under the specific loading conditions applied in this study.

Because the microstructural assessment was qualitative and only two principal processing strategies were compared, a statistically meaningful correlation or regression between microstructural parameters, hardness, case depth and wear cannot be established from the present dataset. Such an analysis would require quantitative microstructural descriptors and a larger number of processing conditions.

The overall analysis of the results therefore indicates that the scanning strategy provides the most favorable compromise between hardness level, hardness profile stability, microstructural homogeneity, and process controllability. Although the single-shot process made it possible to obtain a greater effective case depth, the scanning method allowed the formation of a more homogeneous hardened layer in the zone most important from the point of view of contact fatigue resistance and service durability. This is particularly important in the case of CV joint raceways, where local structural discontinuities may promote stress concentration and crack initiation under cyclic rolling–sliding contact conditions. In addition, the scanning process is better suited to the concept of linear energy input control and thus offers practical possibilities for tailoring the hardened layer in accordance with the predicted distribution of subsurface stresses in components with complex geometry.

Future work will focus on optimizing scanning-based induction hardening parameters to achieve an optimal balance between effective case depth (EDH_500_HV), hardness distribution, and microstructural homogeneity, followed by validation under representative drivetrain loading conditions through component-level and full-system NVH and durability testing.

From an industrial processing perspective, the two hardening strategies differ in the way heat input is controlled. In the single-shot condition, the complete functional zone was heated simultaneously for 28 s, whereas in the scanning condition the coil moved along the raceway at a controlled feed rate of 5 mm·s^−1^. The scanning strategy therefore provides an additional means of controlling local energy input through the combination of generator power and coil feed rate. This enables adjustment of the local thermal cycle and effective case depth without simultaneously exposing the complete functional region to the same thermal history.

Each investigated parameter set was repeated three times, providing an indication of reproducibility within the experimental conditions. However, no formal process capability analysis or large-series production study was performed, and therefore the present results should not be interpreted as a quantitative demonstration of production-scale repeatability.

A direct comparison of total cycle times cannot be made from the present data. The single-shot heating time was 28 s, whereas the total duration of the scanning cycle depends on the axial travel distance and auxiliary machine operations in addition to the feed rate. Consequently, the industrial advantage of the scanning process demonstrated here primarily relates to controllability of local heat input and case-depth tailoring rather than to a demonstrated reduction in total processing time.

The tribological response of both variants remained comparable. The mean steady-state coefficient of friction was 0.49 ± 0.02 for the scanning condition and 0.48 ± 0.01 for the single-shot condition, while the mean specimen mass loss was 0.8 ± 0.1 mg for both treatments. Thus, the improved control of the hardened-layer characteristics obtained with the scanning strategy was achieved without a measurable deterioration in friction or wear behavior under the investigated test conditions.

The present study has several limitations that should be considered when interpreting the results. Only one representative industrial processing condition was evaluated for each hardening strategy, and the microstructural assessment was qualitative rather than quantitative. In addition, circumferential case-depth mapping and production-scale process capability analysis were not performed. The tribological tests were conducted under simplified dry reciprocating sliding conditions with a limited number of independent tests (n = 3 per condition) and therefore do not fully reproduce the lubricated rolling–sliding contact conditions of an operating CV joint. Consequently, the present findings should be regarded as a comparative assessment of the two hardening strategies under the investigated conditions rather than as a complete validation of their full industrial process windows or in-service durability.

Based on these comparative results, the scanning strategy was selected for further process optimization. Future work will investigate the effect of generator power and coil feed rate over a wider parameter range.

## 5. Conclusions

Based on the conducted study on the effect of the induction hardening strategy on hardened layer depth, microstructure, and tribological properties, the following conclusions can be drawn:

1. Both investigated induction hardening strategies, single-shot and scanning, produced a surface layer with predominantly martensitic morphology and hardness exceeding 650 HV1. However, the hardening strategy clearly affected the hardness distribution and effective case depth.
2. The single-shot strategy produced a greater effective case depth, with EDH_500_HV = 3.04 ± 0.15 mm, compared with 2.13 ± 0.02 mm for the scanning strategy (n = 3). At the same time, the scanning-treated condition exhibited a higher near-surface hardness of approximately 700–780 HV1, compared with approximately 670–740 HV1 for the single-shot condition.
3. Qualitative macro- and microstructural observations showed a visually more regular hardened-zone boundary and more uniform microstructural morphology for the scanning condition within the analyzed service-critical cross-sections. The single-shot condition contained local regions differing in contrast and morphology from the surrounding matrix. Because no quantitative stereological analysis or independent phase identification was performed, these observations should not be interpreted as quantitative evidence of microstructural homogeneity, phase constitution, or circumferential case-depth uniformity.
4. Both hardening strategies exhibited comparable tribological behavior under dry reciprocating sliding conditions. The mean coefficient of friction was 0.49 ± 0.02 for the scanning condition and 0.48 ± 0.01 for the single-shot condition, while the mean specimen mass loss was 0.8 ± 0.1 mg for both treatments. Thus, the greater EDH_500_HV obtained with the single-shot strategy did not result in a measurable improvement in friction or wear resistance under the investigated test conditions.
5. Under the investigated conditions, the scanning strategy provided greater flexibility in controlling local energy input and tailoring the effective case depth through the combination of generator power and coil feed rate, while maintaining tribological performance comparable to the single-shot strategy. However, the present study does not provide sufficient data to determine whether these improvements justify the additional process complexity or potential energy demand associated with scanning. A complete industrial assessment would require direct comparison of total cycle time, total energy consumption, process capability, equipment complexity, and large-series production repeatability for both strategies.

## Figures and Tables

**Table 1:** Chemical composition of the investigated C50 steel (wt.%).

Steel Grade	Chemical Composition, wt.%										
	C	Si	Mn	P	S	Cu	Cr	Ni	Mo	Al	Ti
C50	0.50	0.26	0.77	0.017	0.013	0.24	0.21	0.15	0.04	0.03	-

**Table 2:** Induction hardening parameters for single-shot and scanning strategies.

Parameter	Single-Shot Strategy	Scanning Strategy	Unit
HMI power setpoint	23	45	%
Generator power indicated by HMI	50	66	kW
Heating time	28	—	s
Coil feed rate	—	5	mm·s^−1^
Rotational speed	150	150	rpm
Operating frequency	19	19	kHz
Nominal effective coil inner diameter	61	61	mm
Coil-to-part radial lift-off distance	2	2	mm
Quench flow rate	240	240	L·min^−1^

**Table 3:** Tribological test parameters.

Parameter	Value	Unit
Counter-body material	100Cr6	—
Ball diameter	10	mm
Normal load	30	N
Frequency	2	Hz
Half-stroke length, *s*	2.5	mm
Full stroke length, 2*s*	5	mm
Number of cycles	28,800	—
Lubrication condition	Dry	—
Temperature	22 ± 1	°C
Relative humidity	49 ± 3	%

**Table 4:** Average friction coefficients for the investigated material pairings.

Material Pairing (Specimen/Ball)	μ_avg	SD
SCAN HARD/100Cr6	0.49	0.02
SINGLE-SHOT HARD/100Cr6	0.48	0.01

**Table 5:** Average mass loss of the tested specimens and balls. Values are presented as mean ± SD (n = 3). Statistical comparisons between the scanning and single-shot strategies were performed using a two-sided Welch’s *t*-test (α = 0.05).

Material Pairing	Average Specimen Mass Loss [mg]		Average Ball Mass Loss [mg]	
	Δm_p_	SD	Δm_k_	SD
SCAN HARD/100Cr6	0.8	0.1	0.5	0.1
SINGLE-SHOT HARD/100Cr6	0.8	0.1	0.5	0
Welch’s *t*-test, *p*-value	1.000	-	1.000	-

**Table 6:** Averaged measurements of wear tracks on specimens.

Material Pairing	Wear Track Width [mm]	Maximum Wear Track Depth [µm]	Cross-Sectional Area of the Wear Track [µm^2^]
SCAN HARD/100Cr6	1.66 ± 0.06	20.4 ± 2.4	22,533 ± 1692
SINGLE-SHOT HARD/100Cr6	1.70 ± 0.07	19.7 ± 3.1	20,834 ± 3183

**Table 7:** Averaged measurements of wear tracks on balls.

Material Pairing	Average Wear Scar Diameter [mm]	Maximum Relative Height of Irregularities [µm]	Cross-Sectional Area Above the Reference Plane [µm^2^]
SCAN HARD/100Cr6	1.68 ± 0.04	20.6 ± 3.2	22,775 ± 2297
SINGLE-SHOT HARD/100Cr6	1.75 ± 0.03	21.3 ± 1.5	21,753 ± 2720

## Data Availability

The original contributions presented in this study are included in the article. Further inquiries can be directed to the corresponding author.
